# Skin cancer risk in alopecia areata: a systematic review and meta-analysis

**DOI:** 10.3389/fonc.2026.1787992

**Published:** 2026-05-11

**Authors:** Simonetta I. Gaumond, Alireza Abdshah, Isabella Kamholtz, Peyton V. Warp, Keyvan Nouri, Antonella Tosti, Joaquin J. Jimenez

**Affiliations:** 1 Department of Biochemistry and Molecular Biology, Miller School of Medicine, University of Miami, Miami, FL, United States; 2 Dr. Phillip Frost Department of Dermatology and Cutaneous Surgery, Miller School of Medicine, University of Miami, Miami, FL, United States

**Keywords:** alopecia areata (AA), basal cell carcinoma (BCC), melanoma, nonmelanoma skin cancer (NMSC), skin cancer, squamous cell carcinoma (SCC)

## Abstract

**Introduction:**

Skin cancers are the most common malignancy worldwide, and identifying populations with altered risk is essential for informing prevention and surveillance strategies. Emerging evidence suggests that alopecia areata (AA) may be associated with reduced skin cancer risk, potentially reflecting enhanced cytotoxic immune activity.

**Methods:**

We conducted a systematic review and meta-analysis to evaluate the incidence of skin cancers in patients with AA. PubMed, Embase, Scopus, and ClinicalTrials.gov were searched through July 2025. Of 1, 039 records identified, eight studies met inclusion criteria, with six included in the quantitative synthesis.

**Results:**

AA was associated with a statistically significant reduction in melanoma incidence (OR 0.58; 95% CI, 0.36-0.94; p = 0.028). Overall skin cancer risk was reduced but not statistically significant (OR 0.58, 95% CI, 0.27-1.22). Pooled estimates for basal cell carcinoma (OR 0.43; 95% CI, 0.11-1.75) and squamous cell carcinoma (OR 0.66; 95% CI, 0.28-1.57) suggested directionally reduced associations, but did not reach statistical significance. Between-study heterogeneity was high (I^2^ >80% for BCC and SCC), however sensitivity analyses, including leave-one-out tests, confirmed the stability of the protective trend.

**Discussion:**

These findings suggest that AA is not associated with increased skin cancer risk and demonstrates an inverse association with melanoma incidence, although substantial heterogeneity and sensitivity analyses warrant cautious interpretation. These results provide important baseline context for patient counseling and for interpreting longterm safety data as systemic therapies, including JAK inhibitors, are increasingly used.

**Systematic Review Registration:**

https://www.crd.york.ac.uk/prospero/, identifier CRD420251123793.

## 1 Introduction

Skin cancers, including melanoma, basal cell carcinoma (BCC), and squamous cell carcinoma (SCC), are amongst the most common malignancies worldwide and represent a substantial burden for patients and health systems (1–4). Identifying populations with either heightened or reduced risk has important implications for prevention strategies, screening recommendations, and mechanistic insights into tumor biology. Importantly, the scalp is a sun-exposed site, and patients with alopecia may be particularly vulnerable to UV-induced carcinogenesis. In scarring alopecias, chronic inflammation and permanent follicular destruction may create a microenvironment that facilitates keratinocyte carcinogenesis, contributing to an increased incidence of BCC and SCC on the scalp (5). By contrast, alopecia areata (AA) is a non-scarring autoimmune alopecia, and emerging evidence suggests that its immunologic profile may paradoxically be inversely associated with melanoma and other skin cancers. As systemic immunomodulatory therapies for AA expand, understanding disease-specific cancer risk has become increasingly important for patient counseling and long-term safety assessment.

AA is a chronic, autoimmune hair loss disorder with a lifetime prevalence of approximately 2% (6). It is characterized by perifollicular T cell infiltration and disruption of hair follicle immune privilege (7). Unlike many autoimmune diseases, which are typically associated with an increased cancer risk (8, 9), AA may be associated with a reduced incidence of malignancies. This paradox mirrors observations in vitiligo, another autoimmune skin disorder marked by cytotoxic T cell activity, in which multiple studies have demonstrated a lower incidence of melanoma and nonmelanoma skin cancers (NMSC) (10).

Despite these observations, the relationship between AA and skin cancer remains uncertain. Several large population-based studies report significantly decreased incidence of melanoma and NMSC in AA, while others show neutral or even increased risks. This inconsistency leaves clinicians uncertain about the true relationship between AA and skin cancer risks, complicating patient counseling and long-term surveillance.

The recent approval of Janus kinase (JAK) inhibitors for AA underscores the urgency of clarifying this association, as long-term immunosuppression could alter cancer risk profiles. These highly effective therapies modulate immune pathways that may influence cancer susceptibility, yet long-term safety data remain limited (11–13). Understanding the baseline risk of skin cancer in patients with AA is therefore essential for both clinical practice and long-term therapeutic safety.

To address this gap, we conducted a systematic review and meta-analysis of observational studies evaluating the incidence of skin cancers in AA, quantifying risk for melanoma, BCC, and SCC. We aim to clarify the direction and magnitude of risk and contextualize these findings within dermatology, oncology, and immunology.

## 2 Methods

### 2.1 Search strategy and study selection

A systematic review was conducted in accordance with PRISMA guidelines, with the protocol registered in PROSPERO (#CRD420251123793). PubMed, Embase, Scopus, and ClinicalTrials.gov databases from inception through July 2025, using the terms “alopecia areata” AND (“skin cancer” OR “basal cell carcinoma” OR “squamous cell carcinoma” OR “melanoma”). Eligible studies were observational, non-interventional human research reporting incidence of skin cancers in patients with AA compared to non-AA controls. Review articles, conference abstracts without quantitative data, animal studies, interventional trials, and non-English studies were excluded.

**Figure 1:**
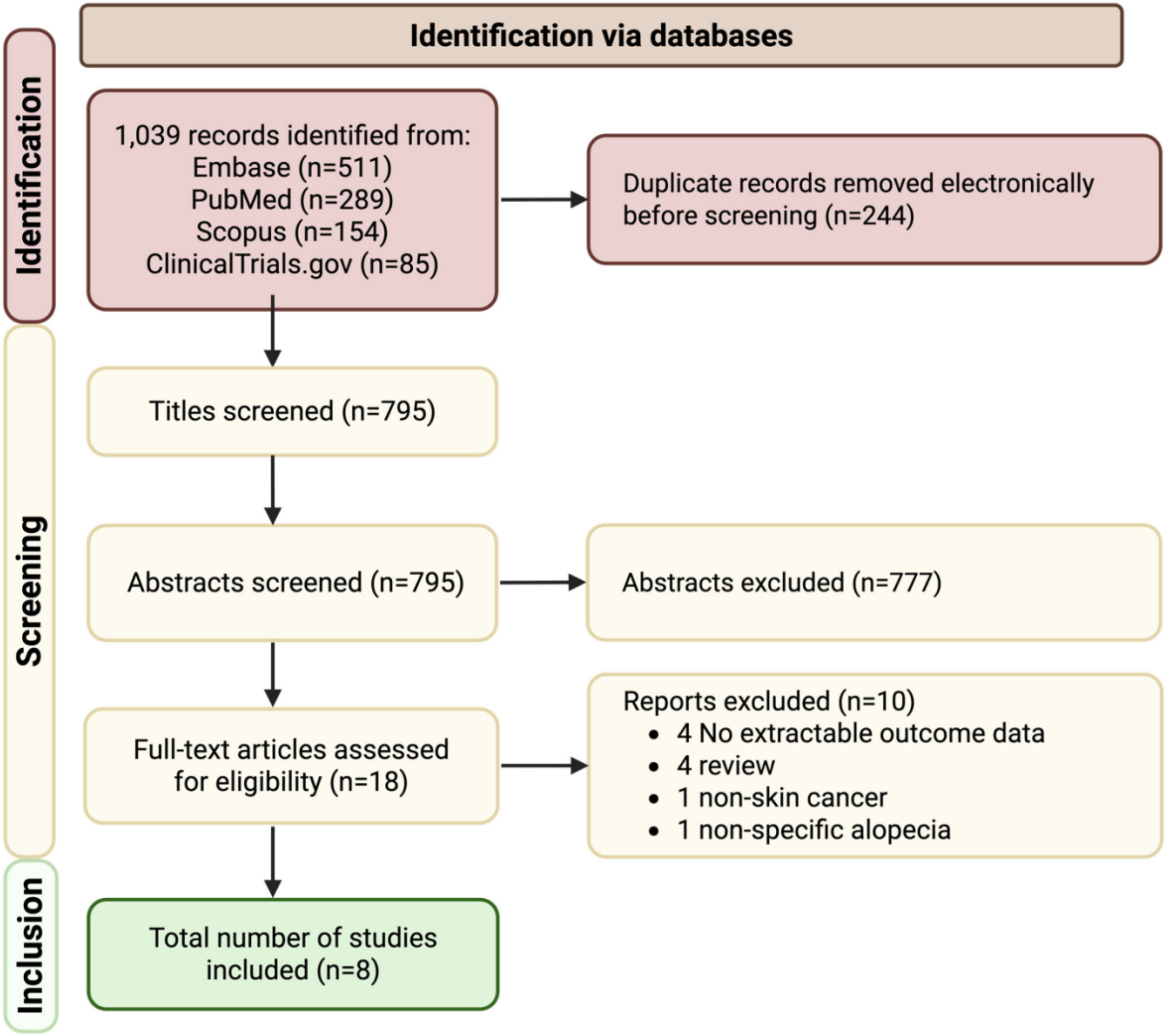
PRISMA flow diagram of study selection. Flow diagram depicting literature search and study selection process. Of 1, 039 records identified across Embase, PubMed, Scopus, and ClinicalTrials.gov, eight studies met inclusion criteria for the systematic review.

A total of 1, 039 records were identified through database searches, of which 244 were duplicates (**Figure 1**). After screening 795 titles and abstracts, 18 articles underwent full-text review. Ten studies were excluded, no extractable outcome data (n = 4), review articles (n = 4), reporting of non-skin cancers (n = 1), or non-specific alopecia (n = 1). Eight studies met inclusion criteria and were included in the systematic review.

### 2.2 Data extraction and measures

Three reviewers (SIG, IK & PVW) independently screened studies, extracted data, and assessed quality. For each study, we recorded cohort sizes, numbers of cancer cases, and reported effect estimates (odds ratios [ORs], hazard ratios [HRs], or standardized incidence ratios [SIRs]) with corresponding 95% confidence intervals (CIs). Outcomes were classified as overall skin cancer or by subtype (melanoma, basal cell carcinoma (BCC), squamous cell carcinoma (SCC)). Discrepancies were resolved by consensus.

### 2.3 Risk of bias assessment

Risk of bias was assessed with the ROBINS-E tool, appropriate for exposure-outcome studies (**Supplementary Table 1**) (14). Two reviewers (SIG & PVW) independently rated seven domains, with consensus on overall judgements (low, moderate, serious, critical).

### 2.4 Statistical analysis

For the meta-analysis, odds ratios (ORs) were calculated directly from raw incidence data whenever possible. Six studies contributed to the pooled estimate for overall skin cancer risk (*Miller* et al., *Mostaghimi* et al., *Conic* et al., *George* et al., *Delcoigne* et al., and *Lee* et al.). For these studies, number of skin cancer cases and cohort sizes were extracted from the published text, tables, or **Supplementary Materials**, and study-specific ORs were derived from these data. Studies reporting hazard ratios (HR), incidence ratios (IR), or standardized incidence ratios (SIR) without sufficient raw incidence data were included in the systematic review but were not pooled in the meta-analysis.

Log-transformed ORs were pooled using random-effects models with restricted maximum likelihood (REML) and Hartung-Knapp adjustment to account for between-study variance. Pooled effect sizes were expressed as ORs with 95% CIs. Heterogeneity was assessed using Cochran’s Q-test and quantified with the I² statistic (15). Subgroup analyses examined melanoma, BCC, and SCC separately. Sensitivity analyses included leave-one-out re-estimation of pooled effects and influence diagnostics. Publication bias was evaluated with funnel plots and Egger’s regression test (16). All analyses were performed in R (version 4.5.1) using the metafor package (version 4.8) (17), and were conducted by AA.

## 3 Results

### 3.1 Systematic review

Eight retrospective studies were included, spanning the United States (18–21), Sweden (22), Denmark (23), Taiwan (24), and Korea (25) (**Table 1**). Sample sizes ranged from just over 500 patients with AA (18, 20) to more than 650, 000 (25), with a mean age of approximately 40 years and a nearly equal distribution of males and females (51.4% female). Five studies included patients with alopecia totalis or universalis (AT/AU) in addition to patchy AA (18, 20, 21, 23, 25). Two studies did not include matched controls (23, 24).

**Table 1:** Characteristics of observational studies included in the systematic review.

Author (year)	Country	Study Design	AA Patients (n)	Controls (n)	AA Subtype(s)	Mean/Median age (% female)	Melanoma outcome (n)		SCC outcome (n)		BCC outcome (n)		Overall skin cancer (%)	
							AA	Control	AA	Control	AA	Control	AA	Control
Miller (2015)	USA	Retrospective study	584	172	AA, AT, AU	35.53 (68.5%)	3	3	3	1	9	6	2.6%	5.8%
Mostaghimi (2016)	USA	Retrospective study	1, 414	4, 242	AA	46.5 (65%)	17	78	18	87	33	194	4.8%	8.5%
Conic (2018)	USA	Retrospective study	563	563	AA, AT, AU	42.47 (74.6%)	2	19	12	50	22	129	6.4%	35.2%
George (2023)	USA	Retrospective study	8, 784	26, 352	AA, AT, AU	45.6 (55.6%)	16	47	52	119	87	238	1.8%	1.5%
Delcoigne (2025)	Sweden	Retrospective study	18, 541	244, 573	AA	35 median (63%)	52	825	65	776	N/A		0.63%	0.65%
Sørensen (2025)	Denmark	Retrospective study	2, 778	0	AA, AT, AU	39 median (63%)	< 5	N/A	< 5	N/A	28	N/A	< 1.3%	N/A
Chen (2018)	Taiwan	Retrospective study	162, 499	0	AA	32.39 (52%)	8	N/A	NMSC				0.023%	N/A
									30		N/A			
Lee (2018)	Korea	Retrospective study	668, 604	3, 343, 020	AA, AT, AU	40.3 (49.1%)	N/A		N/A		N/A		0.0063%	0.0063%

AA, alopecia areata; AT, alopecia totalis; AU, alopecia universalis; BCC, basal cell carcinoma; SCC, squamous cell carcinoma; NMSC, non-melanoma skin cancer. Sample sizes reflect AA patients and controls included in each study. For Sørensen (2025), melanoma and SCC counts <5 were masked per Danish registry policy; overall incidence was estimated as <1.3%. Chen (2018) reported NMSC as a combined category without separate SCC or BCC outcomes.

Several studies reported a lower incidence of skin cancer in AA patients compared to controls. In US cohorts, *Conic* et al. found significantly reduced incidence of melanoma (OR 0.10; 95% CI, 0.02-0.44), BCC (OR 0.14; 95% CI, 0.09-0.22), and SCC (OR 0.22; 95% CI, 0.12-0.42) (all p < 0.001) (20). *Miller* et al. also reported reduced odds of melanoma (OR 0.29; 95% CI, 0.06-1.45), BCC (OR 0.43; 95% CI, 0.15-1.23), SCC (OR 0.88; 95% CI, 0.09-8.44), and NMSC overall (OR 0.47; 95% CI, 0.19-1.15), though only SCC reached statistical significance (p = 0.012) (18). Similarly, *Mostaghimi* et al. observed a 35% reduction in melanoma risk (OR 0.65; 95% CI, 0.39-1.09) and a comparable reduction in NMSC (OR 0.63; 95% CI, 0.48-0.81) (19). In Sweden, *Delcoigne* et al. demonstrated lower hazards for invasive melanoma (HR 0.53; 95% CI, 0.34-0.83), melanoma *in situ* (HR 0.81; 95% CI, 0.56-1.16), SCC (HR 0.65; 95% CI, 0.43-0.98), and SCC *in situ* (HR 0.89; 95% CI, 0.64-1.23) (22). Notably, a nationwide Danish registry reported a markedly decreased incidence of SCC (IR 0.16; 95% CI, 0.04-0.42) and malignant melanoma (IR 0.25; 95% CI, 0.09-0.54), while BCC incidence was neutral but appeared lower in patients with AT/AU (IR 0.89; 95% CI, 0.24-2.29) (23). In Taiwan, *Chen* et al. reported a significantly lower standardized incidence ratio for NMSC (SIR 0.59; 95% CI, 0.38-0.80) (24).

Although the scalp is sun-exposed and theoretically vulnerable in alopecia, few studies reported cancer location in detail. *Delcoigne* et al. noted that, aside from a possibly lower proportion of facial/scalp melanomas, cancer distribution in AA was largely similar to controls (22). *Mostaghimi* et al. also found no significant difference in anatomic location (19). *Conic* et al. provided the most detailed analysis, showing that in AA patients, SCC and BCC were most common on the head/neck and upper extremities, whereas melanomas occurred predominantly on the trunk and extremities (20). Compared with controls, AA patients had a relatively greater proportion of skin cancers on the upper extremities and fewer melanomas localized to the scalp. Collectively, these findings suggest that AA is not associated with a disproportionate increase in scalp-specific cancers, despite loss of hair coverage.

Other findings were more neutral or contradictory. *George* et al. reported no significant differences in melanoma, SCC, and BCC incidence between AA patients and matched controls (21). In Korea, *Lee* et al. found a slightly increased risk of skin cancer in AA overall (HR 1.09; 95% CI, 0.79-1.52), but a decreased risk in AT/AU (HR 0.42; 95% CI, 0.10-1.68) (25). *Chen* et al. also reported an increased risk of melanoma (SIR 1.54; 95% CI, 0.47-2.61) in AA compared to the general population (24).

Risk of bias assessment with ROBINS-E revealed variation across study designs. Single-center cohorts (*Miller, Conic*) were judged at serious risk due to referral populations, unmeasured confounding, and outcome measurement concerns. Large US claims studies (*Mostaghimi, George*) were rated moderate risk, reflecting diagnostic coding limitations and incomplete confounder adjustment. National registries (*Delcoigne, Sørensen, Lee, Chen*) were also rated moderate risk overall: although case ascertainment and representativeness were strong, residual confounding from UV exposure, skin phototype, and treatment history could not be excluded. No study was rated at low overall risk. Overall, most studies were categorized as moderate risk (**Supplementary Table 1**).

### 3.2 Meta-analysis

#### 3.2.1 Overall skin cancer

**Figure 2:**
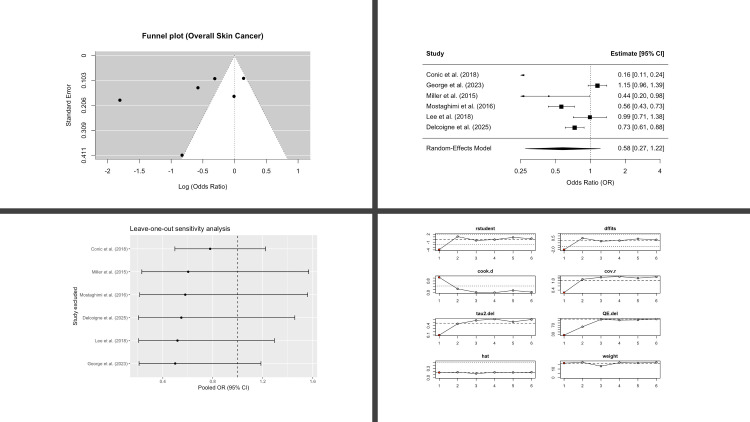
Overall skin cancer in patients with alopecia areata compared with controls. Four-panel figure comprising: (1) Funnel plot of log(OR) versus standard error with pseudo-95% confidence limits to assess small-study effects (2) Forest plot showing study-specific odds ratios and the random-effects (REML, Hartung-Knapp) pooled estimate; squares are proportional to study weight, diamonds show the pooled OR with 95% CI; heterogeneity statistics (I^2^, τ^2^, and Q) are reported. (3) Leave-one-out analysis showing the re-estimated pooled OR after repeatedly excluding each study (vertical dashed line marks the all-studies pooled OR). (4) Egger’s regression assessing small-study effects, displaying the regression line, intercept, and two-sided p-value. OR < 1 indicates lower overall skin cancer risk in AA versus controls.

Six studies contributed to the pooled analysis of overall skin cancer. Patients with AA demonstrated lower odds compared with controls, though the result did not reach statistical significance (OR 0.58; 95% CI, 0.27-1.22; p = 0.12) (**Figure 2**). Between-study heterogeneity was very high (I^2^ = 95%), so we applied REML with Hartung–Knapp adjustment to generate more conservative CIs.

#### 3.2.2 Basal cell carcinoma

**Figure 3:**
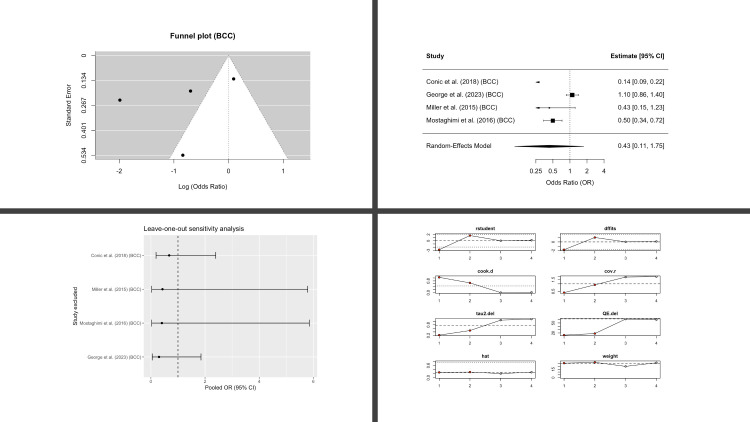
Basal Cell Carcinoma (BCC) in patients with alopecia areata compared with controls. Four-panel figure comprising: (1) Funnel plot of log(OR) versus standard error with pseudo-95% confidence limits to assess small-study effects (2) Forest plot showing study-specific odds ratios and the random-effects (REML, Hartung-Knapp) pooled estimate; squares are proportional to study weight, diamonds show the pooled OR with 95% CI; heterogeneity statistics (I^2^, τ^2^, and Q) are reported. (3) Leave-one-out analysis showing the re-estimated pooled OR after repeatedly excluding each study (vertical dashed line marks the all-studies pooled OR). (4) Egger’s regression assessing small-study effects, displaying the regression line, intercept, and two-sided p-value. OR < 1 indicates lower BCC risk in AA versus controls.

Four studies reported BCC outcomes. The pooled analysis suggested a lower risk of BCC among AA patients, but the estimate was not statistically significant (OR 0.43; 95% CI, 0.11-1.75; p = 0.15) (**Figure 3**). Between-study heterogeneity was very high (I^2^ = 95%).

#### 3.2.3 Squamous cell carcinoma

**Figure 4:**
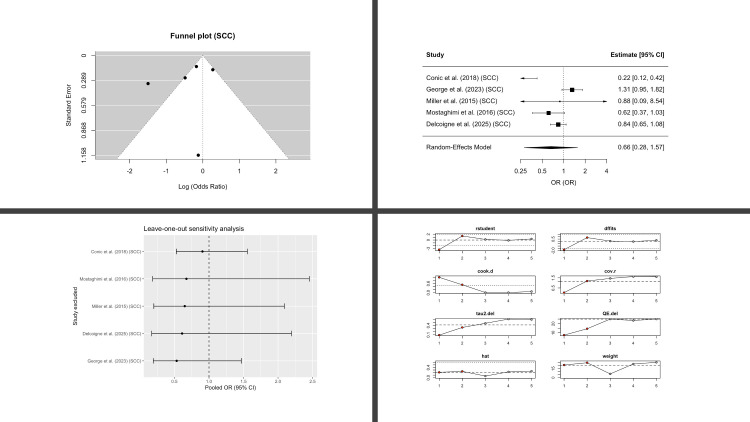
Squamous Cell Carcinoma (SCC) in Patients with Alopecia Areata Compared with Controls. Four-panel figure comprising: (1) Funnel plot of log(OR) versus standard error with pseudo-95% confidence limits to assess small-study effects (2) Forest plot showing study-specific odds ratios and the random-effects (REML, Hartung-Knapp) pooled estimate; squares are proportional to study weight, diamonds show the pooled OR with 95% CI; heterogeneity statistics (I^2^, τ^2^, and Q) are reported. (3) Leave-one-out analysis showing the re-estimated pooled OR after repeatedly excluding each study (vertical dashed line marks the all-studies pooled OR). (4) Egger’s regression assessing small-study effects, displaying the regression line, intercept, and two-sided p-value. *Note: OR < 1 indicates lower SCC risk in AA versus controls.*.

Five studies reported SCC incidence. The pooled estimate showed a directionally consistent reduction in risk that did not reach statistical significance (OR 0.66; 95% CI, 0.28-1.57; p = 0.26) (**Figure 4**). Between-study heterogeneity was high (I^2^ = 84%).

#### 3.2.4 Melanoma

**Figure 5:**
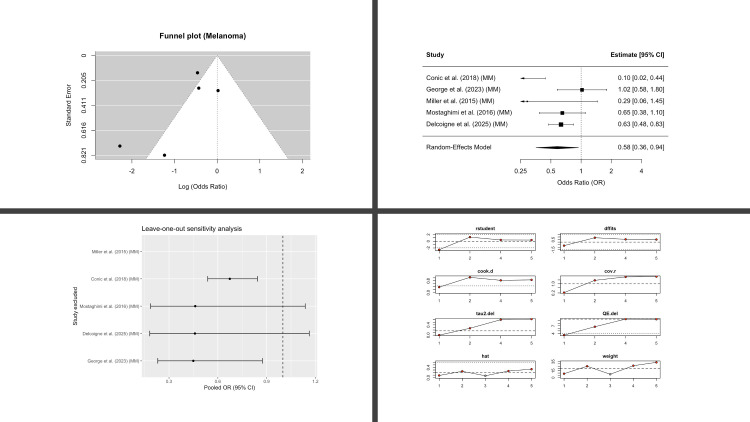
Melanoma in patients with alopecia areata compared with controls. Four-panel figure comprising: (1) Funnel plot of log(OR) versus standard error with pseudo-95% confidence limits to assess small-study effects (2) Forest plot showing study-specific odds ratios and the random-effects (REML, Hartung-Knapp) pooled estimate; squares are proportional to study weight, diamonds show the pooled OR with 95% CI; heterogeneity statistics (I^2^, τ^2^, and Q) are reported. (3) Leave-one-out analysis showing the re-estimated pooled OR after repeatedly excluding each study (vertical dashed line marks the all-studies pooled OR). (4) Egger’s regression assessing small-study effects, displaying the regression line, intercept, and two-sided p-value. OR < 1 indicates lower melanoma risk in AA versus controls.

Five studies contributed melanoma outcomes. In contrast to BCC and SCC, the pooled analysis showed a statistically significant inverse association with melanoma among AA patients (OR 0.58; 95% CI, 0.36-0.94; p = 0.028) (**Figure 5**). Between-study heterogeneity was moderate (I^2^ = 58%). Although statistically significant, this inverse association was sensitive to exclusion of large registry-based cohorts, with attenuation of significance in leave-one-out analyses.

#### 3.2.5 Sensitivity analysis

Across all outcomes, heterogeneity was a prominent feature. Overall skin cancer, BCC, and SCC each demonstrated very high heterogeneity (I^2^ = 84-95%), with little reduction on leave-one-out testing, suggesting that inconsistency was not driven by any single study. In contrast, melanoma showed only moderate heterogeneity (I^2^ = 58%) and more stable estimates, although statistical significance attenuated when certain large cohorts were excluded. These findings indicate that while the direction of effect consistently suggested lower odds of skin cancer in AA, variability between studies warrants cautious interpretation of pooled estimates. No evidence of publication bias was detected on funnel plot inspection or Egger’s regression.

## 4 Discussion

This systematic review and meta-analysis synthesizes available observational evidence examining skin cancer risk in patients with AA and healthy controls. Across multiple cohorts, AA was associated with a statistically significant inverse association with melanoma incidence, with consistent directionally reduced associations for BCC and SCC despite non-significant pooled estimates. Substantial between-study heterogeneity was observed, particularly for keratinocyte carcinomas, reflecting variability in study design, populations, and outcome ascertainment. Given the observational nature of the included studies, these findings should be interpreted as associations rather than evidence of causality.

The immunologic mechanisms discussed below are intended to provide biologic plausibility for the observed epidemiologic patterns and should be regarded as hypothetical frameworks rather than causal inferences derived from this meta-analysis or from the underlying observational studies.

AA is characterized by dense infiltration of autoreactive CD8+ T cells with collapse of hair follicle immune privilege (7). These effector cells produce interferon (IFN)-γ, a cytokine central to tumor immunosurveillance through enhanced antigen presentation and cytotoxic lymphocyte activation (26). Immune pathways implicated in AA therefore overlap with mechanisms involved in antitumor immunity, providing a biologically plausible mechanistic context for the observed inverse association with melanoma (27, 28).

Interleukin (IL)-15 further contributes to this immune milieu by sustaining tissue-resident memory CD8+ T cells and natural killer (NK) cells, both of which play complementary roles in tumor immunosurveillance (29). Unlike IL-2, IL-15 preferentially maintains cytotoxic effector populations without promoting regulatory T cell expansion, favoring persistent immune activation (30, 31). Experimental oncology studies demonstrate that IL-15-driven immune circuits enhance cytotoxic lymphocyte recruitment and tumor control, offering a biologically coherent, though indirect, framework through which AA-associated immune activity may intersect with cancer susceptibility (32–36). NK cells may be particularly relevant in melanoma, given their capacity to target malignant cells that evade immune detection via MHC class I downregulation (37–42).

Collectively, these observations suggest that AA may serve as a human disease model characterized by sustained cytotoxic immune activation, in which IFN-γ- and IL-15-dependent pathways simultaneously drive autoimmune hair follicle destruction and may intersect with pathways relevant to antitumor immunity. While these mechanistic interpretations cannot establish causality from observational data, they provide a coherent biological context for the epidemiologic patterns observed in this meta-analysis and help explain why melanoma, in particular, demonstrates the most consistent protective signal.

From a clinical perspective, these findings provide important context for patient counseling. While chronic autoimmune diseases are often associated with elevated malignancy risk, AA does not appear to share this pattern for skin cancer. Importantly, the data do not support intensified skin cancer surveillance beyond standard population-based recommendations in patients with AA in the absence of additional risk factors. Individualized risk assessment should continue to consider ultraviolet exposure, skin phototype, family history, and prior immunosuppressive therapies.

These observations are particularly relevant in the era of Janus kinase (JAK) inhibitor therapy for moderate-to-severe AA. Regulatory concerns regarding malignancy risk with systemic JAK inhibition in other disease contexts necessitate careful interpretation of long-term safety data in AA (43, 44). Establishing a disease-specific baseline risk is essential for contextualizing future pharmacovigilance findings and distinguishing treatment-related effects from disease-intrinsic immune biology.

Several limitations merit consideration. All included studies were observational, precluding causal inference. The small number of eligible studies limited statistical power and restricted subgroup analyses. Adjustment for key confounders, including cumulative ultraviolet exposure, skin phototype, geographic variation, and treatment history, was inconsistent across studies. The included cohorts spanned diverse geographic regions with differing ultraviolet exposure levels, population skin phototypes, and healthcare systems, which likely contributed to the substantial heterogeneity observed. Several studies combined patchy AA with alopecia totalis or universalis, which may represent biologically distinct phenotypes with differing immune intensity and cancer risk. Moreover, outcome ascertainment relied largely on administrative coding or registry data, introducing potential misclassification. Despite these limitations, sensitivity analyses supported consistent directionality of effect estimates, and no strong evidence of publication bias was detected.

## 5 Conclusion

This systematic review and meta-analysis demonstrates that AA is not associated with an increased risk of skin cancer and rather establishes an inverse association with melanoma incidence. These findings support the concept that AA represents a distinct autoimmune phenotype characterized by heightened cytotoxic immune surveillance, with potential implications for skin cancer risk. By establishing a disease-specific baseline risk, this work provides essential context for patient counseling and for interpreting long-term safety data as systemic therapies, including JAK inhibitors, become increasingly integrated into AA management. Continued prospective and mechanistic studies will be critical to further refine risk stratification and guide evidence-based clinical decision-making.

## Data Availability

The raw data supporting the conclusions of this article will be made available by the authors, without undue reservation.

## Supplementary material

Supplementary Table 1 Risk of Bias Assessment Using ROBINS-E Framework. Risk of bias ratings across seven domains for each included study, classified as low, moderate, serious, or critical. Overall risk of bias was determined according to ROBINS-E guidance: the overall judgment was anchored to the most severe domain-level rating. Clinic-based cohorts were generally rated at higher risk due to potential confounding and outcome measurement bias, whereas registry-based cohorts demonstrated lower overall bias.
